# Initiation of a global consortium to study the progression of age-related macular degeneration: RIMR AMD consortium report # 1

**DOI:** 10.1007/s00417-025-07081-4

**Published:** 2025-12-29

**Authors:** Srinivas R. Sadda, Charles C. Wykoff, Itay Chowers, Jean-Francois Korobelnik, Raja Narayana, Rajeev R. Pappuru, Frank G. Holz, Robyn Guymer, Chui Ming Gemmy Cheung, David S. Boyer, Michael Ip, Heiko G. Niessen, Nancy Holenkamp, Mary Durbin, Stephanie Magazzeni, Anne-Marie Cairns, Carlos Ciller, Natasa Jovic, Joseph Blair, Sandro De Zanet, Aaron Y. Lee

**Affiliations:** 1 RIMR AMD Consortium, Pasadena, CA USA; 2 Doheny Eye Institute, 150 North Orange Grove Blvd, Pasadena, CA 91103 USA; 3 Department of Ophthalmology, University of California - Los Angeles, Los Angeles, CA USA; 4 Retina Consultants of Texas, Houston, TX USA; 5 Hadassah Medical Center, Faculty of Medicine, the Hebrew University of Jerusalem, Jerusalem, Israel; 6 CHU Bordeaux, Service d’ophtalmologie, France; 7 University of Bordeaux, INSERM, BPH, UMR1219, Bordeaux, F-33000 France; 8 Anant Bajaj Retina Institute, L.V. Prasad Eye Institute, Hyderabad, India; 9 Department of Ophthalmology, University of Bonn, Bonn, Germany; 10 Center for Eye Research Australia, Royal Victorian Eye and Ear Hospital, The University of Melbourne, Melbourne, Australia; 11 Singapore National Eye Center, Singapore, Singapore; 12 Retina Vitreous Associates, Los Angeles, CA USA; 13 Boehringer Ingelheim Pharma GmbH & Co. KG, Biberach, Germany; 14 Roche Pharmaceuticals, Basel, Switzerland; 15 Topcon Healthcare Inc, Oakland, NJ USA; 16 Carl Zeiss Meditec AG, Munich, Germany; 17 Optos plc, Dunfermline, Scotland, UK; 18 Ikerian AG, Bern, Switzerland and RetinAI U.S. Inc., Boston, MA USA; 19 University of Washington, Seattle, WA USA

**Keywords:** Age-related macular degeneration, Consortium, OCT, Data sharing

## Abstract

**Purpose:**

To describe the design and organizational structure of a global collaborative consortium aimed at aggregating longitudinal multimodal imaging data to better understand the progression of age-related macular degeneration (AMD) and facilitate therapeutic development.

**Methods:**

The Ryan Initiative for Macular Research (RIMR) AMD Consortium was established as a nonprofit organization, bringing together academic institutions, biopharmaceutical companies, and imaging technology providers. The consortium collects, de-identifies, and harmonizes longitudinal optical coherence tomography (OCT) data, as well as associated clinical metadata, from multiple international clinical centers using a cloud-based infrastructure. Imaging data is converted and stored in DICOM format, and associated clinical data is mapped to the OMOP Common Data Model. All analyses are conducted within a secure cloud environment, supporting both built-in and member-contributed artificial intelligence (AI) tools.

**Results:**

As of the time of reporting, the Consortium has ingested over 100,000 OCT volumes from more than 5,000 subjects across 7 global cohorts spanning 4 continents and 3 major OCT platforms. Based on information provided by the data providers, the dataset encompasses a wide range of AMD stages, from normal aging to late-stage neovascular or atrophic AMD, with longitudinal follow-up extending beyond 15 years for some subjects. A data harmonization pipeline has been established to convert all ingested OCT data to the DICOM standard and is thus ready for automated analysis to gain disease-related insights.

**Conclusions:**

The RIMR AMD Consortium represents a novel model for global collaboration in AMD research, enabling the pooling and analysis of heterogeneous imaging data while addressing privacy, regulatory, and interoperability challenges. This framework may serve as a model for similar initiatives in other ocular diseases.

## Introduction

Despite dramatic therapeutic advances in therapy over the last two decades, age-related macular degeneration (AMD) remains a leading cause of severe irreversible vision loss in older individuals worldwide [1]. To identify and address scientific obstacles and to accelerate the development of AMD therapeutics, the Ryan Initiative for Macular Research (RIMR; formerly termed the Beckman Initiative for Macular Research) program was initiated in 2009. The centerpiece of the RIMR program is an interdisciplinary annual conference that brings together global experts from various clinical and research domains to engage in intense and focused discussions related to advancing therapies for AMD. One of the first outputs of the Beckman/RIMR program was the development of a new universally accessible, clinically-applicable staging scheme for AMD, termed the Beckman scale [2, 3]. The RIMR participants, however also acknowledged the need for a more granular scale which takes full advantage of the rich data available from modern multimodal retinal imaging technologies, and could be used to support more detailed scientific investigation. A major impediment for the development of a more sophisticated staging system, is in large part, the lack of sufficient longitudinal data regarding the progression of AMD from the earliest stages to vision threatening late-stage disease which is necessary to inform such a system.

As a result, one significant conclusion from the work of the RIMR program was that a significant obstacle for the development of novel and early intervention therapeutics for AMD, was this paucity of information regarding the progression of AMD using multimodal retinal imaging, and in particular from optical coherence tomography (OCT). Many researchers worldwide came to a similar conclusion, and a number of programs were initiated in the hope of addressing these deficiencies. One such example is the MACUSTAR program which is a European Union sponsored Industry-Academic partnership and observational clinical study with stated objectives to characterize the functional and anatomic deficits in eyes with intermediate AMD (iAMD), and specifically to develop and validate functional, structural and patient-reported outcome measures for iAMD, in cooperation also with regulatory agencies [4]. To this end, MACUSTAR has successfully recruited 600 subjects with iAMD across seven European countries all of whom have been carefully phenotyped structurally and functionally and are being followed for at least 6 years to define the rate of progression in various biomarkers.

MACUSTAR is already providing invaluable data that will help to shape future AMD clinical trials [5]. There are, however, significant limitations which remain. First, while a sample of 600 subjects is substantial, it may still not capture the true variability of AMD, which itself may represent more than one disease. Second, the MACUSTAR cohort is limited to Europe (which represents < 10% of the world’s population), and thus may not reflect the heterogenous phenotype or progression of AMD worldwide. Indeed, it is already well-established that the appearance of AMD-associated deposits (e.g. drusen) may differ in different populations [6]. Moreover, studies that have compared the rate of progression of AMD-associated outer retinal atrophy, have shown differences between populations of Asian and European ancestral backgrounds [7, 8]. 

Given these significant limitations and knowledge gaps, it has become increasingly apparent that a global longitudinal study with modern imaging technology, in particular OCT, will be critical to advance progress in AMD therapeutics. The assembly of such diverse and high-quality imaging datasets will also be essential for development of high-performing AI models [9, 10]. The time and cost for conducting such a study prospectively, however, is almost certainly prohibitive, particularly since the path to a commercializable product by a sponsor as a direct result of this effort is not immediately apparent. In such a situation, where the value of the information/insights is clear but the cost of acquisition cannot be justified by any single entity, the establishment of a collaborative consortium to share in the costs and benefits is a common, promising solution.

Further, the most cost-effective, timely, and comprehensive approach, would be to collect this data retrospectively from collaborative centers around the world. The data sources for such a collection could be diverse, ranging from epidemiological studies [11], registries, control arms of therapeutic trials, and ophthalmology clinical centers. Such a retrospective collection strategy, however, can have significant limitations as images may be acquired with different devices with varying acquisition protocols and at variable intervals. In addition, associated clinical data will also have similar variability. Normally, such inconsistent data would be difficult to analyze to glean meaningful insights. However, in the era of big data and artificial intelligence, tools are now available which can harness this data and generate useful analytics [12]. 

As an example, AI-based techniques can be used to harmonize or transform data among different devices, and AI algorithms can be used to segment and classify images in a device-agnostic manner [13]. These innovations offer the prospect for the full potential of these large and disparate retrospective datasets to be realized.

Although the technology to utilize these datasets may exist, other significant hurdles remain to be overcome to allow these datasets to be pooled and analyzed. One such problem is the implementation of increasingly restrictive privacy safeguards by governmental agencies. For example, the European Union (EU)’s General Data Protection Regulation (GPDR) limits the movement of patient data outside of the EU territory [14]. Aside from privacy concerns, another challenge to data sharing is concerns over data ownership and the commercial value of data/monetization [15]. Concerns such as these have hindered cooperation and have historically undermined attempts to perform collaborative analyses. To address these limitations, some data scientists have advocated the use of federated approaches [16] where data does not travel to a central location, but rather is analyzed remotely. The federated approach, however, is not without limitations as some remote locations may not have the edge computing processing power to manage the analytical tasks.

To establish a functional framework to facilitate global collaboration towards addressing critical knowledge gaps in the AMD space, while managing concerns that have hindered data sharing, the RIMR AMD Consortium was initiated.

This report summarizes the organizational design and structure of the RIMR AMD Consortium in hope that it might provide guidance or a blueprint for future collaborative programs in other disease states, as well as to make publicly visible this significant initiative, in the event that others wish to join the consortium or seek to access the data to address important questions that are perhaps not yet considered. The results at this stage center around the ingestion and standardization of imaging data from various sources, which provides an important benchmark of the success and viability of the Consortium. Future reports will focus on more detailed results of the analyses.

## Methods

### Mission/objective

The RIMR AMD Consortium was established as a legal entity and a 503(c)(3) non-profit organization incorporated in the State of California in 2023. The initial establishment was by the primary author of this manuscript (SS), who also serves as the Principal Investigator for the RIMR program. As a non-profit organization, the Consortium was granted tax-exempt status allowing all funding to be directed to support the Consortium’s objectives. The stated mission or primary objective of the Consortium is to facilitate the development of therapeutics for patients with AMD (particularly to prevent, stop, or slow development of vision threatening advanced AMD), through the aggregation and analysis of global longitudinal multimodal imaging datasets of patients with AMD, to aid in better defining biomarkers and endpoints for future trials, and facilitate development of a more granular severity scale based on multimodal imaging that predicts progression to severe disease.

### Key concepts

The most critical defining attributes of any consortium are its members, and the basic organizing principle of the RIMR AMD Consortium is an academia/clinician – industry partnership, with academic/clinical institutions providing imaging and de-identified clinical data, biopharma providing major funding, and associate imaging company members providing software development/interfaces to facilitate the extraction of imaging data. The initial or founding academic/clinician members of the Consortium were largely participants in the RIMR annual conference who expressed interest in sharing data and collaborating to establish this enterprise. These initial members who comprised the steering committee of the consortium then identified additional potential members based on geographic diversity, ready availability of datasets which could be shared (a minimum of 100 AMD subjects, ideally with at least one year of follow-up were deemed to be necessary for inclusion), and willingness to comply with the consortium’s master agreement.

There were a number of additional key guiding principles or concepts which guided the organization of the Consortium. First, for the Consortium to be of true value, a Global Data Collection was deemed to be necessary. This was thought to be vital as most existing datasets/studies are within a specific region. As AMD is a global disease and affects populations with variable ethnic backgrounds and environmental exposures, it likely has different manifestations around the globe, with differing progression characteristics. These differences have implications for a better understanding of the disease progression and for the designs of trials. By collecting datasets from around the world these regional differences will be able to be studied.

A second key concept was that imaging studies would serve as the core or organizing kernel of data around which the Consortium would be built. While it is certainly desirable to aggregate both imaging and clinical (e.g. demographics, ocular comorbidities, visual function) data, imaging data is attractive as it is objective and amenable to automatic verification and analysis. For example, using imaging, it is possible to definitively determine that a patient truly has AMD (i.e. the correct diagnosis) based on identification of typical drusen and other features of AMD. In addition, many existing national registries such as the American Academy of Ophthalmology (AAO) Intelligent Research In Sight (IRIS) [17] or Vestrum Health Database [18], lack imaging data at scale, and thus the Consortium’s data collection would address the most pressing gap. However, whilst imaging data is held to be the centerpiece, the Consortium’s intention is still to acquire all available clinical data whenever possible. To be included as a quantum of data in the Consortium, the minimum dataset was defined to be an OCT volume from a single visit. OCT data was deemed to be the most valuable imaging modality as it is volumetric and highly amenable to automated quantitative analysis with a wide array of existing tools.

A third critical concept was cloud-based storage and analysis. All data were planned to be uploaded securely and stored in a de-identified fashion in the cloud. While servers could theoretically be anywhere in the world, the ultimate location(s) of the servers would be dictated by regional data sharing regulations. For the initial deployment, the Consortium chose to maintain the servers within the European Union (Germany). Critically, in addition to storage, all analytical processes were required to be executed in the same local cloud as well. Thus, in the Consortium’s design, data would not move to a remote analysis center outside of the EU cloud region.

A final key concept of the Consortium that follows naturally from the storage and analytical architecture is data ownership and access. As highlighted in the Introduction, a major concern with any consortium effort is data ownership. The cloud-based storage and analysis plan obviates many of these concerns, since data ownership is retained at all times by the data provider and only access for Consortium-approved analyses is granted by the data provider. Data providers are also given the ability to withdraw their data from the Consortium at any time and are free to share their own data outside of the Consortium in any manner they deem appropriate.

### Governance

A legal document or Consortium Master Agreement (CMA) was developed based on these guiding principles and the founding members were required to sign the agreement for the Consortium to be launched.

The Governance structure of the Consortium, as defined in the CMA, was centered around a Steering Committee, which holds decision-making authority for the consortium. The Steering Committee members consisted of three types of founding members: Industry Members: (1) Biotech/Pharma Companies specializing in developing and marketing AMD therapeutics (primarily responsible for funding the Consortium’s operational costs), (2) Academician/Clinician Members (primarily responsible for contributing imaging data to Consortium Database), and (3) Associate Members: Imaging Technology Companies specializing in ophthalmology and AMD (primarily responsible for contributing the necessary software tools to facilitate acquisition and analysis of imaging data from the clinical centers). Although the Associate Members were not granted voting rights, their participation and input in the Steering Committee discussions were thought to be essential. Examples of the types of decisions taken on by the Steering Committee in their governance role include selection/admittance of new Consortium members, selection/confirmation of operational staff/officers, and approval of Consortium-wide analyses.

Although all decision-making power was granted to the Steering Committee, the actual execution of Consortium tasks and activities required an Operations team that was to be appointed by the Steering Committee. This Operations team was to include senior management (Executive Director, Director of Operations, Chief Administrator) as well as the technical team (database developers, data scientists) and the annotations team (image readers/graders). The Steering Committee also granted the Operations team the freedom to select 3rd party vendors in order to execute on certain functions required to achieve the Consortium’s mission.

### Principles of data harmonization and standardization

To be as inclusive as possible, the Consortium welcomed the submission of imaging data from any vendor, including multiple OCT device manufacturers. Although a DICOM standard has been developed for OCT, and a number of efforts are underway to encourage compliance with these DICOM standards [19, 20], much of the retrospective data that will be targeted for ingestion by the Consortium will be stored in proprietary industry formats.

To manage this challenge, the Consortium Operations team contracted a third party vendor who custom-built a Consortium-specific version of their commercial image management platform to remotely ingest data from the clinical centers, anonymize the data as needed, and make it available within the platform for viewing and analysis using tools available in the platform. In addition, all ingested image data was also exported as DICOM files into a Consortium-managed Amazon Web Services (AWS) S3 bucket, and thus available for analysis using other tools [21]. 

**Fig. 1:**
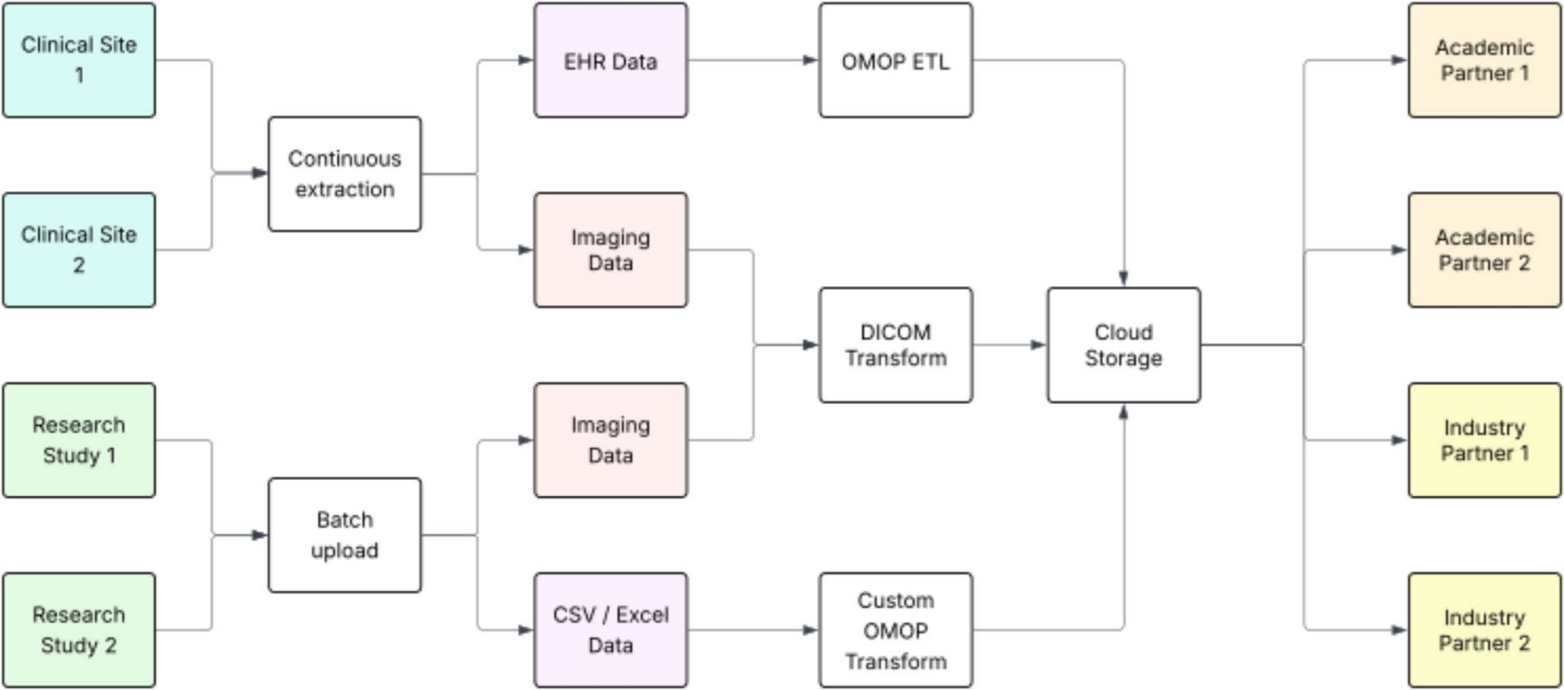
Schematic diagram illustrating the flow of imaging and clinical data from the the clincial centers to the cloud storage environment of the Consortium. After transformation of all imaging data to the DICOM standard and all clinical data to the OMOP standard it is made available for analysis within the cloud computing environment by the consortium academic and industry partners

As noted above, while imaging data was the principal focus, associated clinical metadata was also deemed to be of value and is also being collected and organized into the Observational Medical Outcomes Partnership (OMOP) Common Data Model (CDM) [22]. By converting all imaging data to DICOM and collecting all clinical data in OMOP, the Consortium approach also aligns well with the US National Institutes of Health Bridge2AI program which aims to create data sets that are properly documented and ready for use with AI technologies [23]. The flow of data to the Consortium is illustrated in the schematic diagram in Fig. 1.

### Collaborative research and Cloud-based analyses

In addition to contributing to the database, it was the intention of the RIMR AMD Consortium that Consortium members and their respective research teams would contribute to the analysis of the Consortium data. A structure was established for individual Consortium members to submit project proposals for review by the Steering Committee, and upon approval, would be authorized to complete these analytical projects. The results of these analyses, which will initially focus on the progression of AMD in different regions, will be the subject of future reports. Consortium members, however, are required to adhere to the rules of the consortium requiring all analytics to be run in the cloud as no mechanism was created to allow images to be downloaded.

Such analyses will be able to be conducted in one of three ways: (1) utilize integrated artificial intelligence (AI)-based analytical tools available in the 3rd party image database platform; (2) connect a member’s own AI-based analytical tool into the 3rd party platform using the Medical Open Network for AI (MONAI) standard [24]; or (3) directly operate on the DICOM files in the cloud using their own analytical tools. In the third case, each of the consortium members who wish to do analytics on the dataset would be contractually obligated to peer a virtual private cloud for all the compute and storage necessary in the same cloud region as the dataset.

While AI-based automated tools are preferred because of the volume of expected data in the Consortium database, human annotation and manual analytics can also be performed. Regardless, human expert verification of a portion of the automatically analyzed data is a component of the intended Consortium analytical plan. Specifically, the Consortium will contract certified human graders from an established reading center to review the classifications and quantitative outputs from ~ 1% of the collected data. The precise percent of human-verified data will be adjusted upwards or downwards based on the observed performance. Errors in automatic grading detected by the human graders will be used as needed to refine model training.

## Results

**Fig. 2:**
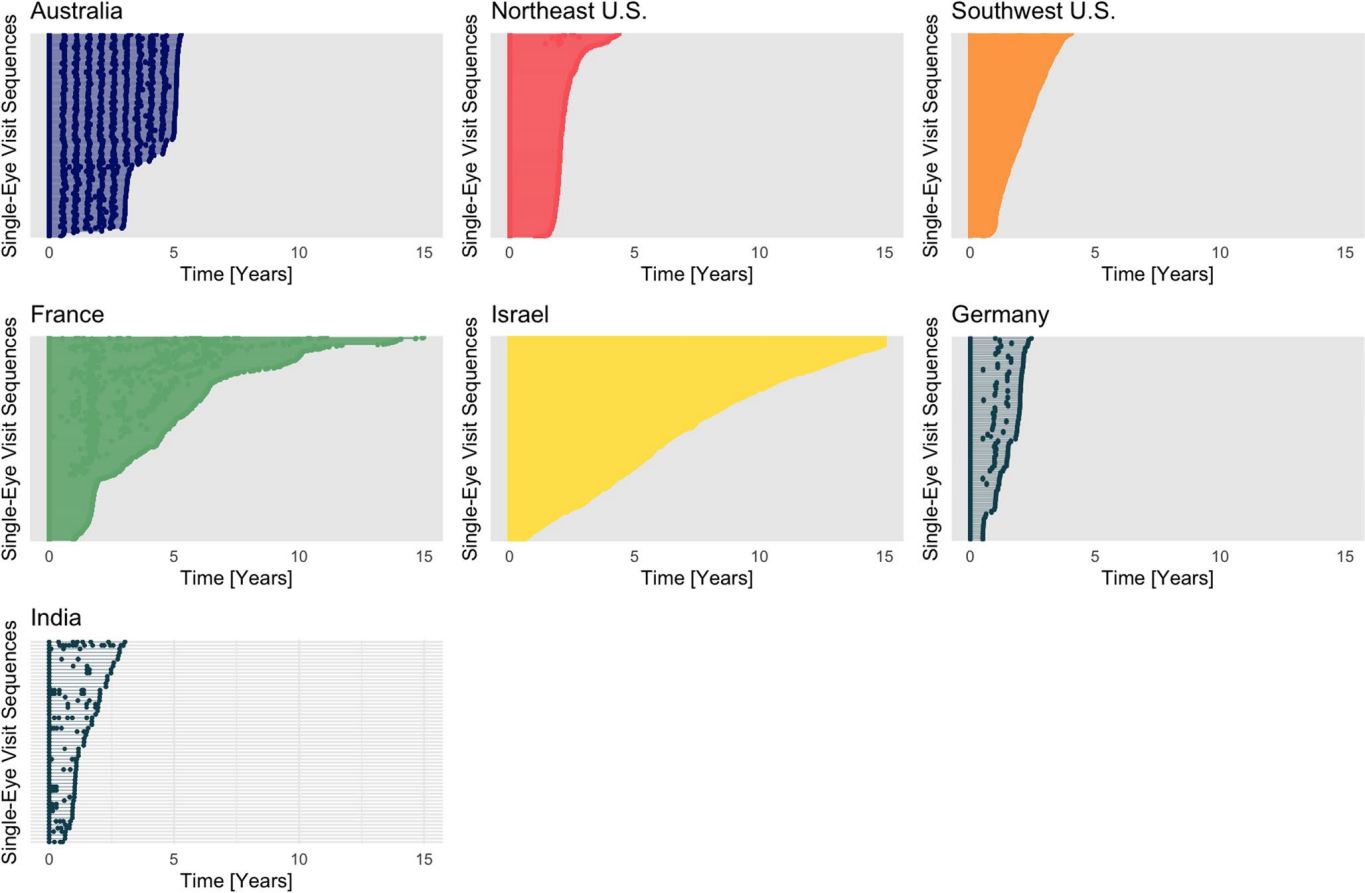
Overview of Longitudinal Data Collected by Location. Bar graph plots illustrating the OCT imaging data included in the Consortium database to date. Each dot along the Y-axis represents a subject. The number of unique subjects in the datasets range from the smallest, 141 (Australia) to the largest, 1662 (Northwest U.S.). Each dot along X-axis represents a follow-up visit(s) for a patient over time. The shortest follow-up interval is 2 years, whereas the longest follow-up interval is more than 15 years. Eyes with less than 6 months of follow-up are not shown in this plot

After a Consortium-specific customized version of the image management platform was brought online, longitudinal OCT data was successfully ingested into the Consortium database from 7 distinct cohorts across 4 continents. As shown in Table 1; Fig. 2, these include datasets from the Southwestern United States (“Southwest U.S.”),Northeastern United States (“Northeast U.S.”), Germany, France, Israel, India, and Australia. Each dot along the Y-axis of Fig. 1 represents an individual subject. Thus far, OCT data from three devices (Zeiss Cirrus OCT, Heidelberg Spectralis OCT, Topcon Triton OCT) has been collected. Table 2 summarizes the distribution of OCT data from different OCT devices at the time of manuscript submission.

**Table 1:** Consortium data by location

Geography	Australia	France	Germany	India	Israel	Southwest USA	Northeast USA	TOTAL
N Subjects	140	810	182	233	1253	1176	1671	5465
N Eyes	280	1607	310	368	2492	2351	3329	10,738
N Visits (Mean (SD))	9.21 (2.22)	2.44 (1.47)	1.76 (0.89)	1.61 (1.46)	27.4 (32.8)	2.96 (1.03)	1.39 (0.49)	8.0 (19.0)
Follow Up Years (Mean (SD))	4.20 (1.12)	3.13 (1.56)	0.68 (0.85)	0.31 (0.67)	5.68 (4.69)	2.03 (0.94)	0.63 (1.05)	2.54 (3.37)
N Subjects with ≥ 3 visits	138	331	54	27	971	724	38	2268
N Subjects with 1 visit only	0	293	97	167	170	38	1198	1963

**Table 2:** Consortium data by OCT device

Device	OCT Volumes
*Heidelberg Spectralis*	104,524 (91.99%)
*Heidelberg HSF-OCT-103*	2012 (1.77%)
*Zeiss 4000*	6505 (5.73%)
*Topcon Triton*	573 (0.50%)
TOTAL	113,614 (100%)

The detailed characteristics of the cohort will be described in future reports describing specific image-based analyses. Future analyses will also report on the frequency of the various stages of AMD across the different cohorts, but based on information gathered from the data providers, the aggregated cohort includes subjects with normal aging, early and intermediate AMD, neovascular AMD, and atrophy.

Figure 1 also highlights the variability of longitudinal follow-up data available, as dots along the X-axis represent patient visits over time. It is apparent that follow-up data ranges from 2 years to more than 15 years, with a mean interval between visits of 133 (+/- 244) days. Multiple OCT exams are available for many of the patients that have long-term follow up.

## Discussion

This first report from the RIMR AMD Consortium summarizes the organizational concepts and principles that have led to the initial collection and harmonization of a global dataset of images (largely OCT) in AMD. These principles are of importance as they allowed a diverse group of clinical centers and industry to come together in a collaborative fashion to tackle a significant problem and knowledge gap for the broader community.

Through 12 months of the Consortium’s operation, over 100,000 OCT volumes have been successfully ingested, from over 5,000 subjects, 7 different locations, with some subjects having follow up extending over 15 years. De novo collection of such long-term data would be prohibitively expensive and virtually impossible to collect in the context of prospective studies, and would take years to collect prospectively at such a scale. The RIMR data should be invaluable for studying AMD disease progression, particularly from very early to late stages of disease.

While the Consortium has launched to a promising start, the initiation phase has uncovered a number of challenges. While conversion from various proprietary standards to a DICOM format has gone relatively smoothly, variability among centers in how exported imaging data is anonymized or organized has created challenges in coupling the imaging data with the clinical data. Streamlining the ingestion process to reduce the burden on the contributing clinical centers is another problem area that will require attention. Finally, certain regions such Africa, East Asia, and Central/South America are not currently well-represented in the Consortium dataset and will be the focus of future membership recruitment efforts.

The solutions to these and other problems will be detailed in future reports. Regardless, we believe that the knowledge gained in the process of bringing this Consortium online will be of value not only to the ophthalmic community but also the broader medical scientific community. The lessons learned in the process of harmonizing and organizing Consortium data may provide guidance going forward to clinical centers/data providers regarding how to collect and store their data to facilitate future data sharing projects. For example, there are a number of other conditions such as diabetic retinopathy, myopia, inherited retinal degenerations, and inflammatory diseases which could potentially benefit from this global data collection and analysis to provide novel disease insight and the disease variability across the world and association with systemic diseases. It is our hope that the lessons learned from the RIMR AMD Consortium may benefit such future efforts.
